# TRAF6 neddylation drives inflammatory arthritis by increasing NF-κB activation

**DOI:** 10.1038/s41374-018-0175-8

**Published:** 2019-01-09

**Authors:** Kewei Liu, Kaizhe Chen, Qian Zhang, Lianfang Zhang, Yufei Yan, Changjun Guo, Jin Qi, Kai Yang, Fei Wang, Ping Huang, Lei Guo, Lianfu Deng, Changwei Li

**Affiliations:** 1 Shanghai Key Laboratory for Prevention and Treatment of Bone and Joint Diseases with Integrated Chinese-Western Medicine, Shanghai Institute of Traumatology and Orthopedics, Ruijin Hospital, Shanghai Jiaotong University School of Medicine, 197 Ruijin 2nd Road, 200025 Shanghai, China; 2 Department of Orthopedic Surgery, Guanghua Integrative Medicine Hospital, No.540 Xinhua Road, 200052 Shanghai, China; 3 Department of Orthopedics, The First Affiliated Hospital of Soochow University, Suzhou, Jiangsu Province China

**Keywords:** Neddylation, Phosphoinositol signalling

## Abstract

Neddylation is a process similar to ubiquitination, and is critical in various inflammatory diseases; however, its importance in the pathogenesis of inflammatory arthritis is not well understood. Here, we investigated the role of neddylation in collagen-induced arthritis (CIA) and its clinical relevance. We showed that neddylation-related genes, including *NEDD8* and *CULLIN*-1, were significantly upregulated in inflamed arthritic synovia. Functionally, neddylation activation was crucial for synovitis of CIA, as the inhibition of neddylation by MLN4924 significantly suppressed synovial cell proliferation and inflammatory responses. Mechanistically, neddylation mediated inflammatory arthritis by regulating NF-κB activation in fibroblast-like synovial cells (FLSs). Furthermore, TNF receptor-associated factor 6 (TRAF6) neddylation at Lys124 was essential for IL-17A-induced NF-κB activation. Replacing the Lys-124 residue with Arg (K124R) resulted in significantly impaired conjugation of NEDD8 to TRAF6, as well as markedly attenuated IL-17A-induced NF-κB activity. Therefore, the pathogenic role of neddylation in CIA as well as its mechanism of action demonstrated here provides a new insight into understanding the role of post-transcriptional modifications in the arthritis inflammatory response.

## Abstract (web summary)

The role of neddylatin in inflammatory arthritis is not well understood. Here we reveal that NEDD8 and CULLIN-1 are significantly upregulated in the synovium of patients with RA. Neddylation activation is crucial for the pathobiology of collagen-induced arthritis. Mechanistically, TRAF6 neddylation at Lys124 is essential for IL-17A-induced NF-κB activation in synoviocytes.

## Introduction

Rheumatoid arthritis (RA), an autoimmune and systemic inflammatory disorder characterized by chronic synovitis and cellular infiltration, often leads to bone erosion and cartilage destruction [1]. Significant therapeutic advances have greatly improved the lives of patients with RA over the last few decades [2]. In particular, treatment strategies targeting various cellular and molecular aspects of the immune system, such as B cells, T-cell co-stimulation, Janus kinase (Jak)-mediated cytokine signaling, and the cytokines tumor necrosis factor-α (TNF-α) and interleukin-6 (IL-6), have proven to be highly effective in limiting synovial joint inflammation as well as preventing irreversible joint and cartilage destruction and hyper-proliferation of synovial cells [3]. Despite this progress, not all patients with RA respond to these therapies or they become resistant to these therapies over time [4]. Thus, advancing the understanding of the underlying etiology is required for the development of innovative therapies for treating RA.

Posttranslational modification by ubiquitin has been demonstrated to be a critical mechanism regulating the pathogenesis of inflammatory arthritis [5]. The role of particular modifications, such as phosphorylation in the NF-κB pathway, and their role in the arthritis inflammatory response have been extensively studied [6]. The ubiquitin proteasome system (UPS) controls NF-κB activity by regulating the degradation of the inhibitory protein IκB, which results in the expression of many pivotal cytokine and chemokine mediators that contribute to the inflammation response in RA, including TNF-α, IL-6, IL-8, inducible nitric oxidase synthase, and cyclooxygenase-2 (COX-2) [7]. In preclinical animal models, inhibition of UPS effectively ameliorates arthritis symptoms by limiting the inflammatory response and suppressing synovium hyperplasia [5, 8]. Thus, the ubiquitin proteasome pathway could be a target for the treatment of inflammatory arthritis.

Cullin-RING E3 ligases (CRLs) are a class of ubiquitin ligases that control the proteasomal degradation of numerous target proteins, including IκB [9]. Except for ubiquitination, the activity of CRLs is positively regulated by the conjugation of an NEDD8 polypeptide onto cullin proteins in a process called neddylation [10, 11]. It is clear that NEDD8 modification of the Cul-1 component of ubiquitin ligase enzyme complex SCF^β-TrCP^ is important for the function of SCF^β-TrCP^ in ubiquitination of IkBα and NF-κB precursor p105 [12, 13]. Neddylation occurs through a series of enzymatic reactions similar to those involved in ubiquitination, including the E1 (NAE1/UBA3) and E2 (UBC12) reactions [14]. The well-characterized substrates that are modified by NEDD8 include the cullin subunits of CRLs; this modification of cullins is critical for the transfer of ubiquitin from E2-ubiquitin to the substrate [15, 16]. Recent studies have shown that the pharmacological agent MLN4924 can potently inhibit NEDD8-activating enzymes, thereby prohibiting the transfer of NEDD8 onto target proteins [9]. Except for cancer cell survival [17], the neddylation pathway has been demonstrated to play a critical role in regulating the functions of B cells [18], T cells [14], myeloid leukemia cells [19], macrophages [9], and endothelial cells [20] during inflammatory responses. Therefore, neddylation inhibitors could be used as potential anti-inflammatory agents. However, the role of neddylation in inflammatory arthritis is not well known.

Given that CRLs play a key role in the etiology of inflammatory arthritis, and that their activity is also positively regulated by neddylation, here we investigated whether neddylation is involved in the pathogenesis of CIA. Our findings uncover a pathogenic role of neddylation in CIA and delineate a previously unknown mechanism involved in its process.

## Results

### Neddylation is activated in inflamed arthritic synovia

**Fig. 1:**
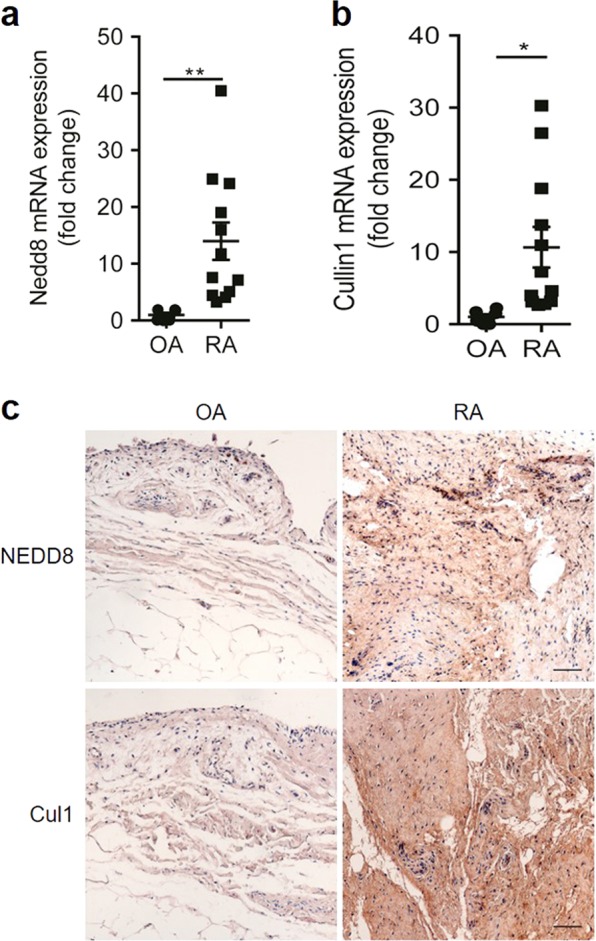
Neddylation activation-related genes expression is increased in inflamed arthritic synovia. **a**, **b** Quantification analysis of *Nedd8* and *Cullin-1* mRNA expression in synovium of OA (*n* = 6) and RA (*n* = 12). **c** immunohistochemical staining analysis of NEDD8 and Cullin-1 expression in synovium of OA (*n* = 6) and RA (*n* = 12). *P < 0.05, **P < 0.01. *P* values were analyzed by two-tailed *t* tests. Scale bar represents 50 μm

To explore the role of neddylation in inflammatory arthritis, we first identified neddylation-related genes that were expressed in synovial tissues collected from 12 patients who underwent total knee arthroplasty (TKA) surgery for end-stage RA. Furthermore, synovial tissues isolated from six patients with noninflamed osteoarthritis (OA) who underwent TKA were used as control [21]. RT-PCR revealed that mRNA levels of *NEDD8* and *CULLIN-1* were notably increased in the synovium of patients with RA compared to that in patients with noninflamed OA (Fig. 1a, b). Moreover, the upregulated expression of NEDD8 and CULLIN-1 in the inflamed arthritic synovia was further confirmed by immunohistochemical staining analysis (Fig. 1c). Collectively, these data demonstrated that inflammatory arthritis increased neddylation in the synovium.

### Inhibition of neddylation reduces the severity of collagen-induced arthritis

**Fig. 2:**
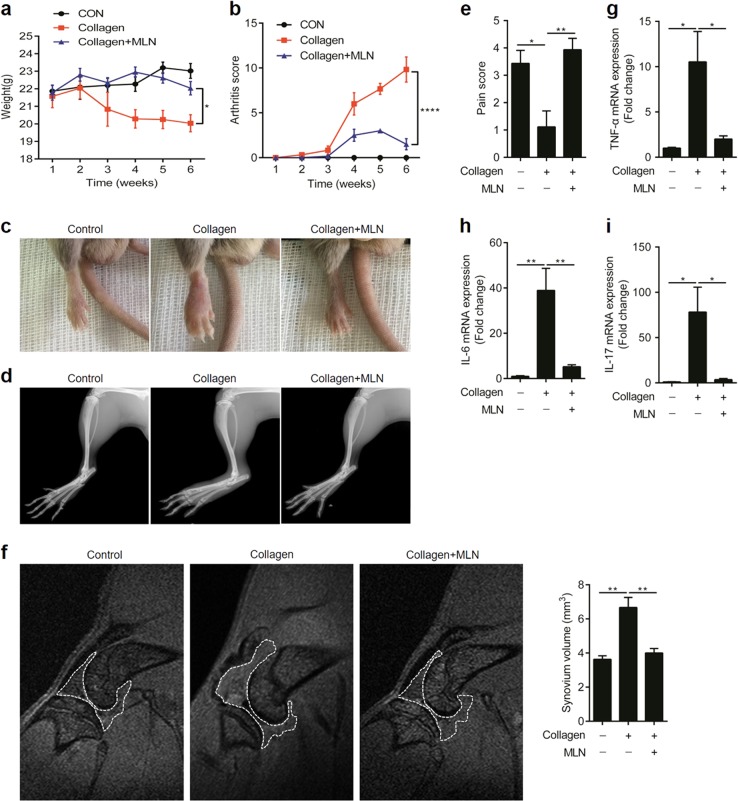
Inhibition of neddylation reduces the severity of collagen-induced arthritis. **a**, **b** Time curve of body weight and arthritis score. **c**, **d** Visual inspection and X-ray analysis of affected hind limbs. **e** Pain score of affected paws. **f** Synovium volume detected by MRI of the knee joints. **g**–**i** Quantification analysis of TNF-α, IL-6, and IL-17A mRNA expression in the affected paws. *P < 0.05, **P < 0.01, ****P < 0.0001. *P* values were analyzed by two-way ANOVA in (**a**, **b**) and one-way ANOVA in (**e**–**i**)

Next, we investigated the pathogenic role of neddylation in the CIA model of RA. MLN4924 was used to attenuate neddylation in this model. As shown in Fig. 2a, inhibition of neddylation by MLN4924 markedly suppressed the weight loss induced by the intradermal injection of type II collagen. Likewise, administration of MLN4924 significantly ameliorated severe swelling, erythema, and joint rigidity in the hind paws, as indicated by arthritis scores (Fig. 2b), visual inspection (Fig. 2c), X-ray analysis (Fig. 2d), and pain scores (Fig. 2e). Furthermore, MLN4924 treatment resulted in a notably milder synovial hyperplasia, as evidenced by the decreased synovium volume detected by magnetic resonance imaging (MRI) of the knee joints (Fig. 2f). In addition, the reduced expressions of TNF-α, IL-6, and IL-17A in the paws of the CIA model further confirmed the protective role of MLN4924 in CIA (Fig. 2g–i). Taken together, these results demonstrated that inhibition of neddylation reduced the severity of CIA.

### Inhibition of neddylation suppresses IL-17A-induced synovial cell proliferation and inflammatory responses

**Fig. 3:**
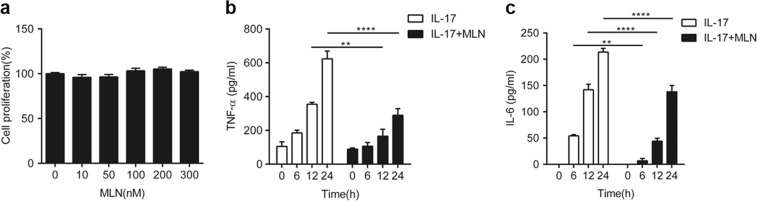
Inhibition of neddylation suppresses IL-17A-induced inflammatory responses. **a** Synovial cell proliferation rate detected by CCK8 assay with different doses of MLN4924 stimulation. **b**, **c** Quantification analysis of TNF-α and IL-6 secretion in the culture medium of synovial cells induced by 100 ng/ml IL-17A with or without 100 nM MLN4924 pretreatment. **P < 0.01, ****P < 0.0001. *P* values were analyzed by two-way ANOVA in (**a**) and one-way ANOVA in (**b**, **c**)

**Fig. 4:**
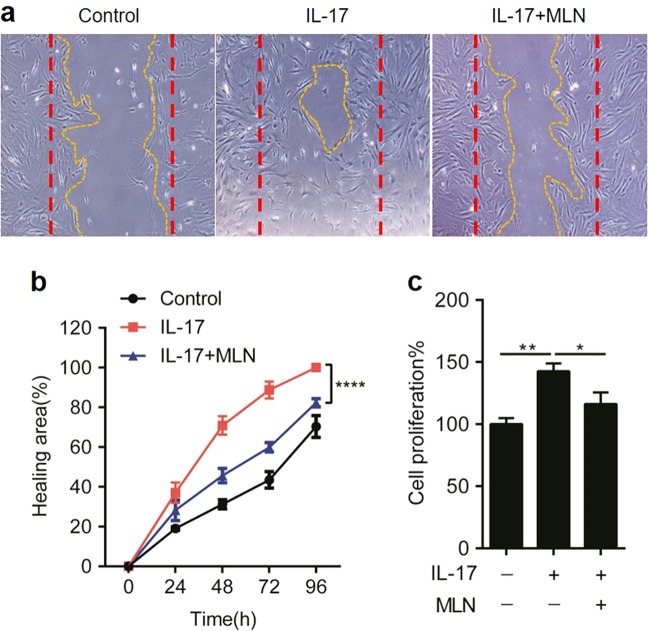
Inhibition of neddylation suppresses IL-17A-induced synovial cell proliferation. **a**–**c** Synovial cells were incubated with 100 ng/ml IL-17A with or without 100 nM MLN4924, and the proliferation was detected by scratch wound healing (**a**, **b**) and CCK8 assay (**c**). The red dotted line represents the area of the scratch at *t* = 0 h. The yellow dotted line represents the area of the scratch at *t* = 72 h.*P < 0.05, **P < 0.01, ****P < 0.001. *P* values were analyzed by two-way ANOVA in (**b**) and one-way ANOVA in (**c**)

Since IL-17 plays a pathogenic role in the CIA model of RA [22], we next evaluated the effects of inhibiting neddylation on IL-17A-induced synovial cell proliferation and inflammatory responses. As shown in Fig. 3a–c, 100 ng/ml IL-17A induced IL-6 and TNF-α expression in a time-dependent manner in human primary synovial cells; however, these processes were significantly attenuated by treatment with 100 nM MLN4924 (Fig. 3a–c). Not surprisingly, the proliferative effect of IL-17A on human primary fibroblast-like synovial cells (FLSs) was abundantly restrained by MLN4924 administration (Fig. 4a–c). Therefore, these data demonstrated that inhibition of neddylation suppressed IL-17A-induced synovial cell proliferation and inflammatory responses in vitro.

### Neddylation regulates IL-17A-induced synovial cell proliferation and inflammatory responses via the NF-κB pathway

**Fig. 5:**
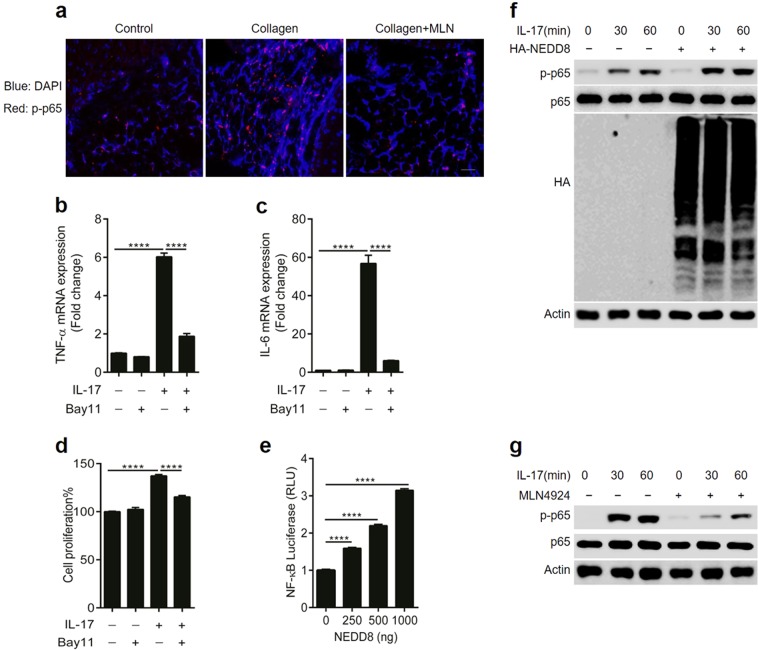
Neddylation regulates IL-17A-induced synovial cell proliferation and inflammatory responses via NF-κB pathway. **a** Immunofluorescence staining of phosphorylated p65 in synovium of mice with CIA. **b**, **c** Quantification analysis of TNF-α and IL-6 mRNA expression in synovial cells induced by 100 ng/ml IL-17A with or without 10 μM BAY11 pretreatment. **d** Synovial cell proliferation rate detected by CCK8 assay. **e** NF-κB transcription activity induced by different doses of NEDD8 in cell line HEK293T. **f** Western blot analysis of p65 phosphorylation in synovial cells induced by 100 ng/ml IL-17A with or without nedd8 transfection. **g** Western blot analysis of p65 phosphorylation in synovial cells induced by 100 ng/ml IL-17A with or without 100 nM MLN4924 pretreatment. ****P < 0.0001. *P* values were analyzed by two-way ANOVA in (**b**, **c**) and one-way ANOVA in (**d**, **e**). Scale bar represents 50 μm

Having observed the role of neddylation in the development of CIA, we next sought to uncover the underlying mechanisms by which neddylation mediated synovitis. NF-κB has been previously identified as a central mediator in the pathogenesis of inflammatory arthritis [6], and our present study also revealed than MLN4924 treatment significantly dampened NF-κB activation in synovium of CIA (Fig. 5a). Furthermore, our present study revealed that inhibition of the NF-κB pathway with the specific inhibitor BAY11 significantly decreased IL-17A-induced expression of IL-6 and TNF-α expression as well as FLS proliferation (Fig. 5b–d). Therefore, we hypothesized that neddylation might regulate the development of inflammatory arthritis by regulating NF-κB activation. To test this hypothesis, NEDD8 was overexpressed in cell line HEK293T, which led to increased NF-κB activity in a dose-dependent manner (Fig. 5e). Furthermore, NEDD8 overexpression enhanced IL-17A-induced p65 phosphorylation (Fig. 5f) in human primary synovial cells. Interestingly, MLN4924 pretreatment also markedly decreased the IL-17A-induced activation of NF-κB (Fig. 5g). Taken together, these results demonstrated that inhibition of neddylation suppresses IL-17A-induced synovial cell proliferation and inflammatory responses.

### TRAF6 neddylation positively regulates IL-17A-induced NF-κB activation

**Fig. 6:**
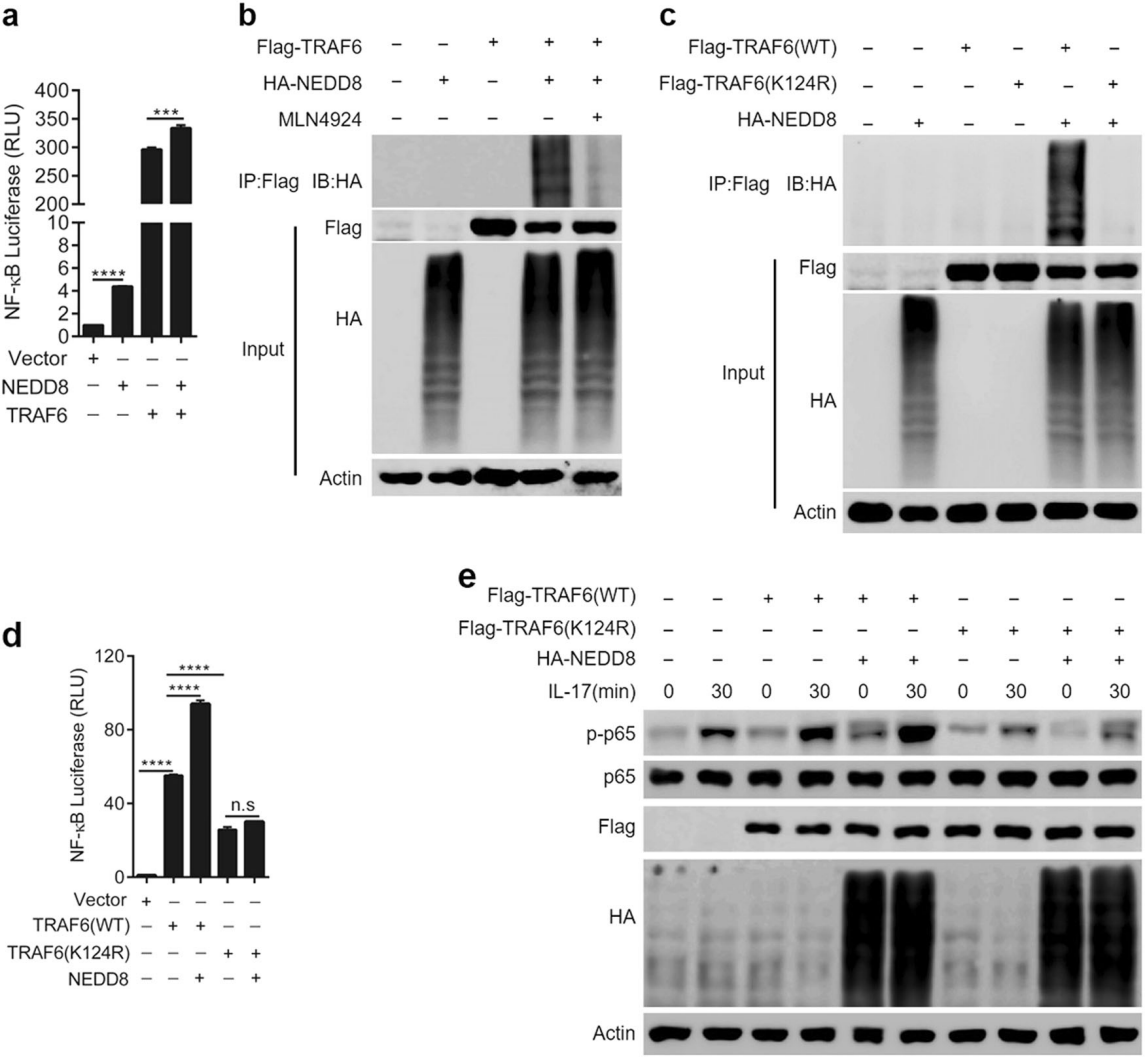
TRAF6 neddylation positively regulates IL-17A-induced NF-κB activation. **a** NF-κB transcription activity induced by NEDD8 and/or TRAF6 transfection in cell line HEK293T. **b** Immunoprecipitation analysis to detect NEDD8 and TRAF6 conjunction in cell line HEK293T. **c** TRAF6-K124R impaired the conjunction of NEDD8 to TRAF6 in cell line HEK293T. **d** K124R mutant of TRAF6 dampened NF-κB transcription activity in cell line HEK293T. **e** K124R mutant of TRAF6 decreased IL-17A-induced p65 phosphorylation in human primary FLSs. ***P < 0.001, ****P < 0.0001. n.s. represents no significance. *P* values were analyzed by two-way ANOVA in (**a**) and one-way ANOVA in (**e**)

TRAF6 is a key adaptor downstream of the IL-17A pathway, and a previous study demonstrated that TRAF6 polyubiquitination was essential for NF-κB activation [23]. However, whether other posttranslational modifications of TRAF6 involved in NF-κB activation remain unknown. Since our present study revealed that NEDD8 and TRAF6 had a synergetic effect in promoting NF-κB activity (Fig. 6a) in cell line HEK293T, we hypothesized that TRAF6 may be neddylated, which then positively regulates NF-κB activation. To test this hypothesis, HEK293T cells were transfected with HA-labeled NEDD8 and FLAG-labeled TRAF6. Immunoprecipitation analysis was then performed, which revealed that multiple NEDD8 molecules were conjugated to TRAF6 (Fig. 6b). After treatment with MLN4924, the level of neddylation on TRAF6 decreased significantly (Fig. 6b). Interestingly, we further found that Lys124 is the predominant NEDD8 acceptor site in TRAF6, and TRAF6-K124R significantly impaired the NEDD8 conjunction to TRAF6 (Fig. 6c). Furthermore, the TRAF6-K124R mutant markedly attenuated the activation of NF-κB signaling (Fig. 6d) as well as IL-17A-induced p65 phosphorylation (Fig. 6e), which further confirmed that Lys124 was the main NEDD8 acceptor site in TRAF6. Taken together, these results demonstrated that TRAF6 neddylation positively regulated IL-17A-induced NF-κB activation.

## Discussion

Ubiquitination has been previously identified as a critical process regulating the pathogenesis of inflammatory arthritis [5]; however, other types of post-translational modifications involved in the immune signaling transduction of this disease needed further investigation. Here, we observed that neddylation-related genes, including *NEDD8* and *CULLIN-1* were significantly upregulated in the synovium of patients with RA compared to that in patients with noninflamed OA. Our data demonstrated that activation of neddylation is crucial for the CIA response. Mechanistically, neddylation mediated inflammatory arthritis by regulating NF-κB activation. Furthermore, TRAF6 neddylation at Lys124 was essential for IL-17A-induced NF-κB activation in FLSs. Therefore, elucidating the pathogenic role of neddylation in CIA and its mechanism of action provides a new insight into the role of post-transcriptional modifications in innate immunity. These findings also offer potential therapeutic strategies for inflammatory arthritis, such as targeting neddylation activation.

Understanding the mechanisms that control responses to inflammation is important for developing effective therapies for inflammatory arthritis. NF-κB is a transcriptional activator that plays a critical role in the pathogenesis of RA [6]. With inflammatory cytokines such as IL-17A, inhibitory proteins such as IκBa are rapidly phosphorylated by IKKa/b, ubiquitinated, and rapidly degraded by the proteasome. This allows for NF-κB to be released from IκB and translocated to the nucleus to activate target genes including TNF-α, IL-6, IL-8, MMPs, and COX-2 [24]. Subsequently, this process results in the recruitment of leukocytes into the joints to maintain chronic inflammation, induces the proliferation of synovial fibroblasts as well as cartilage and bone degradation in the development of arthritis [25]. Therefore, therapeutics that target the NF-κB pathway may provide an effective strategy for treating inflammatory arthritis.

Besides ubiquitination, a number of reports has demonstrated the essential role of neddylation in regulating NF-κB activity. Studies have shown that neddylation exert critical functions in various inflammatory diseases such as atherogenesis [26], colitis [27], and acute lipopolysaccharide-induced endotoxemic shock [20] by regulating NF-κB activity. However, the role of neddylation in the etiology of inflammatory arthritis is not well known. Here, we found that neddylation activation was upregulated in the inflamed arthritic synovia. Furthermore, the loss-of-function experiment demonstrated that by blocking the activation of neddylation with MLN4924, symptoms of CIA including the hyperproduction of inflammatory cytokines and synovial hyperplasia were reduced. In addition, our data also revealed that inhibition of neddylation attenuates the CIA response by decreasing NF-κB activation in the synovium.

Although the well-characterized substrates of NEDD8 modification are the cullin subunits of CRLs, non-cullin substrates of neddylation have also been recently identified for their roles in various signaling pathways. For instance, MYD88 neddylation negatively regulates MYD88-dependent NF-κB signaling by antagonizing its ubiquitination [28]. Furthermore, neddylation of BCA3 promotes its interaction with the p65 subunit and inhibits NF-κB activation [29]. However, whether other pivotal proteins in the NF-κB signaling pathway undergo neddylation remains unknown.

In the present study, we found that TRAF6 neddylation was essential for IL-17A-induced NF-κB activation. TRAF6 is one of seven closely related TRAF proteins, which are adapter proteins linking the TNF receptor superfamily to intracellular signaling transmission [30]. Furthermore, TRAF6 is a key adaptor for the IL-1 receptor/TLR (IL-1R/TLR) superfamily signaling pathway, which serves crucial roles in the proliferation and proinflammatory effects of fibroblast-like synoviocytes (FLSs) [31]. Recent biochemical evidence has indicated that TRAF6 possesses ubiquitin ligase activity that controls the activation of IKK and NF-κB. Furthermore, TRAF6 is auto-ubiquitinated via Lys63 linkages, which depends on an intact RING and the dimeric E2 enzyme UBC13/UEV1A [23]. Inhibiting the TRAF6−UBC13 interaction with a small molecule inhibitor counteracts NF-κB signaling and inflammatory responses in CIA model of RA [32]. However, whether other translational modifications of TRAF6 are essential for NF-κB activation still need to be further elucidated. Here, we demonstrated that TRAF6 is a substrate of NEDD8, and the NAE inhibitor MLN4924 significantly blocked the conjunction of TRAF6 and NEDD8. Furthermore, we found that Lys124 is the main NEDD8 acceptor site in TRAF6, which has been previously reported as the TRAF6 ubiquitination site for IKK activation after IL-1 stimulation [23]. We also found that TRAF6 neddylation at Lys124 was critical for IL-17A-induced NF-κB activity, as the TRAF6-K124R mutant markedly attenuated the IL-17A-induced p65 phosphorylation. Thus, these data identified TRAF6 as a new NEDD8 substrate in regulating NF-κB activity.

Since IL-17 plays a critical role in the development of CIA [22], here we used IL-17A to induce synovial cell proliferation and inflammatory responses in vitro. Besides IL-17R, TRAF6 is also essential for signal transduction from multiple other receptors implicated in inflammatory arthritis, including TNFR, IL-1R, and CD40 [25]. Although dual blockade of TNF-α and IL-1β, or IL-1β and IL-17A reduces murine arthritis, spontaneous skin infections were observed [33]. Thus, partial inhibition of neddylation with a small molecule, such as MLN4924, may be a novel and reasonable approach for treating inflammatory arthritis.

However, we would like to point out some potential limitations of our study. First, although our results revealed that TRAF6 neddylation was essential for IL-17A-induced NF-κB activation, these results were mainly acquired based on in vitro experiments. Whether the same mechanisms are shared in vivo still need further investigations. Furthermore, it must be noted that the types of human arthritis most closely linked with IL-17 are spondyloarthritides [34, 35]. Therefore, our results might be more relevant to ankylosing spondylitis and psoriatic arthritis than RA. Second, TRAF6 neddylation is observed in cells overexpressing Flag-TRAF6 and HA-NEDD8 in the present study. We had sought to determine whether the endogenous TRAF6 was modified by NEDD8 after IL-17A stimulation in human synovial cells, but it might due to too low content, no neddylation modification was detected with mass spectrometry assay.

In conclusion, our study demonstrated that neddylation plays a pathogenic role in CIA. TRAF6 neddylation might mediate inflammatory arthritis responses by regulating NF-κB activation. These findings provide new insight into understanding post-transcriptional modifications in innate immunity as well as offer potential therapeutic strategies for the treatment of inflammatory arthritis, such as targeting neddylation activation.

## Materials and methods

### Synovium tissues collection

Synovium tissues were obtained from 6 patients with noninflamed OA and 12 patients with RA who underwent total joint arthroplasty (TKA) surgery. All human sample acquisitions were approved by the ethical committee of Ruijin Hospital, SJTU School of Medicine, China, and performed in accordance with the Declaration of Helsinki Principles. All participants provided written informed consent, which was obtained before enrollment in the study.

### Mice

Eight-week-old male DBA/1 mice were purchased from Model Animal Research Center of Nanjing University. The animals that were randomly grouped were not performed in a blinded manner. The animals were housed with free access to water and rat diet in an airconditioned room with a 12-h light–dark cycle, at 21−23 °C and 60% relative humidity in the animal facility at Ruijin Hospital, Shanghai Jiaotong University (SJTU) School of Medicine, China. All animal experiments were performed according to the protocol approved by the SJTU Animal Care and Use Committee and in direct accordance with the Ministry of Science and Technology of the People’s Republic of China on Animal Care guidelines. All surgeries were performed under anesthesia and all efforts were made to minimize suffering.

### Collagen-induced arthritis and MLN4924 treatment

Collagen-induced arthritis was performed according to previous report [36]. Briefly, chick collagen type II (CII) (Chondrex, Inc) was dissolved in 10 mM of acetic acid to a 2-mg/ml concentration and emulsified with a complete Freund’s adjuvant (CFA, Sigma-Aldrich). At the beginning of the experiments (day 0), the mice were immunized with a 0.2-ml emulsion containing 100 μg of collagen at the tail base. At 21 days after the first immunization, mice were again challenged with collagen/CFA nearby the primary injection site intradermally. Starting from the day of immunization (d0), some DBA/1 mice were intraperitoneally injected with 30 mg/kg MLN4924 (MedChemExpress) [37] every 3 days. The development of arthritis was assessed by examining the clinical severity in each paw every week by two blinded observers. The clinical severity was quantified according to a graded scale from 0 to 4, as follows: 0, no swelling; 1, swelling in one digit or mild edema; 2, moderate swelling affecting several digits; 3, severe swelling affecting most digits; and 4, the most severe swelling and/or ankylosis. A total arthritis score per mouse was determined by summing the scores of all four extremities.

### Magnetic resonance imaging performance and data analyses

MRI performance and data analysis to evaluate the synovium volume in vivo were performed according to a previous report [38].

### Primary synovial cells isolation and culture

Human specimens were taken with ethical approval of the Shanghai Ruijin Hospital review board. Synovium was obtained from the articular capsule of informed patients, who suffered from end-stage arthropathy (RA) undergoing TKA. As described previously [39], after washing and removing of excess adipose tissue, synovium was dissected into pieces as possible, followed by digestion in 2 mg/ml collagen type I for 4 h, 37 °C. Dispersed synovial cells were cultured in Dulbecco’s modified Eagle’s medium containing 10% fetal bovine serum, 100 U/ml penicillin, 100 U/ml streptomycin, under standard culture conditions. For optimal cell purity and cellular viability, synovial fibroblasts with fourth to sixth passage were used for further experiment.

### Luciferase reporter assay

HEK293T cells were seeded in 24-well plates and transfected the following day by lipofectamine 2000 according to the manufacturer’s instruction. Approximately 100 ng of NF-κB luciferase reporter plasmid and 10 ng of Renilla luciferase reporter plasmid were transfected together with indicated expression plasmids. Luciferase activity was measured 24 h after transfection with Dual Luciferase reporter assay system (Promega). Relative NF-κB activity was calculated as firefly luminescence relative to Renilla luminescence.

### Immunoprecipitation and neddylation analysis

HEK293T cells were seeded in six-well plates and transfected the following day by Lipofectamine 2000 according to the manufacturer’s instruction. Two micrograms of HA-tagged NeDD8 expression plasmid or/and 2 μg of Flag-tagged WT TRAF6 or mutant TRAF6 expression plasmid were transfected. IP assay was performed 36 h after transfection. Briefly, HEK293T cells were lysed in lysis buffer (50 mM Tris-HCl, pH 7.4, 150 mM NaCl, 5 mM Ethylene Diamine Tetraacetic Acid (EDTA), 1% Triton X-100, 10% Glycerol and protease inhibitor cocktail) and the supernatant were incubated with anti-Flag antibody overnight at 4 °C. Then the protein A + G beads were added to the supernatant incubating for another 2 h. After extensive washing, the beads were boiled at 100 °C for 10 min.

### Immunohistochemistry staining analysis

The paraffin-embedded tissues were used for the immunohistochemical analysis of NeDD8 and Cullin1 expression in the synovial tissue from patients and mice. Full-thickness specimens were processed for immunohistochemical analysis as previously described. Briefly, after the slides were incubated with blocking serum (Vectastain ABC Kit; Vector Laboratories, Inc., Burlingame, CA, USA) for 60 min, they were blotted and then overlaid with the primary antibody against Nedd8 and Cullin1 for 2 h at room temperature, respectively. Subsequently, biotinylated secondary antibodies were added into the sections, followed by a peroxidase-labeled streptavidin-biotin staining technique (DAB Kit, Invitrogen, Paisley, UK).

### Scratch wound healing assay

Human primary FLSs were seeded in 12-well plates. When cell confluence reaches 90%, cells were then scratched per well vertically with the P1000 pipette tip to make a scratch. Following washing twice with PBS, cells were treated with 100 ng/ml IL-17A in the presence or absence of 100 nM MLN4924. The same fields were photographed and the scratched area was calculated by Image-pro Plus 6.0.

### Evaluation of pain with paw/foot withdrawal threshold in mice

The paw/foot withdrawal threshold was effective to quantify the severity of affected paws pain in CIA [40]. The mice were placed for acclimation at least 10 min before testing. Subsequently, calibrated Von Frey filaments (Stoelting, Wood Dale, IL, U.S.) were applied perpendicular to the plantar surface of the paws with sufficient bending force for 3–5 s. The positive reaction was defined as a brisk movement with or without lipping or biting. When the mice had a positive reaction, a sequential lower stimulus (smaller filament) was applied. In contrast, in the event of negative response, a greater stimulus (the next largest filament) was used. There was a 1-min interval between every two stimuli. The level of withdraw threshold was calculated and the average of two hind-limb’ scores was recorded.

### Real-time quantitative RT-PCR

Real-time RT-PCR specific primers were used to evaluate gene expression. RNA analysis was done as previously reported [41]. Real-time PCR primer for IL-6: 5′-CTGCAAGAGACTTCCATCCAGTT-3′ (forward) and 5′-GGGAAGGCCGTGGTTGTC-3′ (reverse); TNF-α: 5′-TCAAGGACTCAAATGGGCTTTC-3′ (forward) and 5′-TGCAGAACTCAGGAATGGACAT-3′ (reverse); IL-17A: 5′-GCTCCAGAAGGCCCTCAGA-3′ (forward) and 5′-CTTTCCCTCCGCATTGACA-3′.

### Protein detection by ELISA

The culture medium supernatant was harvested and assayed for cytokine content using commercially available ELISA reagents for TNF-α and IL-6 (Duoset R&D Systems, Abingdon, UK).

### Statistical analysis

All data representative of three independent experiments are present as mean ± SEM. We used two-tailed *t* tests to determine significances between two groups. We did analyses of multiple groups by one- or two-way ANOVA with Bonferroni post-test of GraphPad prism version 6. For all statistical tests, we considered *P* value < 0.05 to be statistically significant.

## References

[1] Wang Q, Sun X (2017). Recent advances in nanomedicines for the treatment of rheumatoid arthritis. Biomater Sci.

[2] Firestein GS (2003). Evolving concepts of rheumatoid arthritis. Nature.

[3] van Hamburg JP, Tas SW (2018). Molecular mechanisms underpinning T helper 17 cell heterogeneity and functions in rheumatoid arthritis. J Autoimmun.

[4] Smolen JS, Aletaha D, McInnes IB (2016). Rheumatoid arthritis. Lancet.

[5] Palombella VJ, Conner EM, Fuseler JW (1998). Role of the proteasome and NF-kappaB in streptococcal cell wall-induced polyarthritis. Proc Natl Acad Sci USA.

[6] Makarov SS (2001). NF-kappa B in rheumatoid arthritis: a pivotal regulator of inflammation, hyperplasia, and tissue destruction. Arthritis Res.

[7] Chitra S, Nalini G, Rajasekhar G (2012). The ubiquitin proteasome system and efficacy of proteasome inhibitors in diseases. Int J Rheum Dis.

[8] Ahmed AS, Li J, Ahmed M (2010). Attenuation of pain and inflammation in adjuvant-induced arthritis by the proteasome inhibitor MG132. Arthritis Rheum.

[9] Chang FM, Reyna SM, Granados JC (2012). Inhibition of neddylation represses lipopolysaccharide-induced proinflammatory cytokine production in macrophage cells. J Biol Chem.

[10] Watson IR, Irwin MS, Ohh M (2011). NEDD8 pathways in cancer, Sine Quibus Non. Cancer Cell.

[11] Kamitani T, Kito K, Nguyen HP, Yeh ET (1997). Characterization of NEDD8, a developmentally down-regulated ubiquitin-like protein. J Biol Chem.

[12] Read MA, Brownell JE, Gladysheva TB (2000). Nedd8 modification of cul-1 activates SCF(beta(TrCP))-dependent ubiquitination of IkappaBalpha. Mol Cell Biol.

[13] Amir RE, Iwai K, Ciechanover A (2002). The NEDD8 pathway is essential for SCF(beta -TrCP)-mediated ubiquitination and processing of the NF-kappa B precursor p105. J Biol Chem.

[14] Jin HS, Liao L, Park Y, Liu YC (2013). Neddylation pathway regulates T-cell function by targeting an adaptor protein Shc and a protein kinase Erk signaling. Proc Natl Acad Sci USA.

[15] Saha A, Deshaies RJ (2008). Multimodal activation of the ubiquitin ligase SCF by Nedd8 conjugation. Mol Cell.

[16] Duda DM, Borg LA, Scott DC (2008). Structural insights into NEDD8 activation of cullin-RING ligases: conformational control of conjugation. Cell.

[17] Soucy TA, Smith PG, Milhollen MA (2009). An inhibitor of NEDD8-activating enzyme as a new approach to treat cancer. Nature.

[18] Milhollen MA, Traore T, Adams-Duffy J (2010). MLN4924, a NEDD8-activating enzyme inhibitor, is active in diffuse large B-cell lymphoma models: rationale for treatment of NF-{kappa}B-dependent lymphoma. Blood.

[19] Swords RT, Erba HP, DeAngelo DJ (2015). Pevonedistat (MLN4924), a First-in-Class NEDD8-activating enzyme inhibitor, in patients with acute myeloid leukaemia and myelodysplastic syndromes: a phase 1 study. Br J Haematol.

[20] Ehrentraut SF, Kominsky DJ, Glover LE (2013). Central role for endothelial human deneddylase-1/SENP8 in fine-tuning the vascular inflammatory response. J Immunol.

[21] Derer A, Bohm C, Grotsch B (2016). Rsk2 controls synovial fibroblast hyperplasia and the course of arthritis. Ann Rheum Dis.

[22] Nakae S, Nambu A, Sudo K, Iwakura Y (2003). Suppression of immune induction of collagen-induced arthritis in IL-17-deficient mice. J Immunol.

[23] Lamothe B, Besse A, Campos AD (2007). Site-specific Lys-63-linked tumor necrosis factor receptor-associated factor 6 auto-ubiquitination is a critical determinant of I kappa B kinase activation. J Biol Chem.

[24] Li Y, Wang LM, Xu JZ (2017). Gastrodia elata attenuates inflammatory response by inhibiting the NF-kappaB pathway in rheumatoid arthritis fibroblast-like synoviocytes. Biomed Pharmacother.

[25] Kim EY, Moudgil KD (2017). Immunomodulation of autoimmune arthritis by pro-inflammatory cytokines. Cytokine.

[26] Asare Y, Ommer M, Azombo FA (2017). Inhibition of atherogenesis by the COP9 signalosome subunit 5 in vivo. Proc Natl Acad Sci USA.

[27] Ehrentraut SF, Curtis VF, Wang RX, Saeedi BJ, Ehrentraut H, Onyiah JC, et al. Perturbation of neddylation-dependent NF-kappaB responses in the intestinal epithelium drives apoptosis and inhibits resolution of mucosal inflammation. Mol Biol Cell. 2016;27:3687–94 10.1091/mbc.E16-05-0273 PMC5170552 27682585

[28] Xie L, Yu S, Wang Z (2017). Nicotinamide adenine dinucleotide protects against spinal cord ischemia reperfusion injury-induced apoptosis by blocking autophagy. Oxid Med Cell Longev.

[29] Gao F, Cheng J, Shi T, Yeh ET (2006). Neddylation of a breast cancer-associated protein recruits a class III histone deacetylase that represses NFkappaB-dependent transcription. Nat Cell Biol.

[30] Bradley JR, Pober JS (2001). Tumor necrosis factor receptor-associated factors (TRAFs). Oncogene.

[31] Wu H, Arron JR (2003). TRAF6, a molecular bridge spanning adaptive immunity, innate immunity and osteoimmunology. Bioessays.

[32] Brenke JK, Popowicz GM, Schorpp K (2018). Targeting TRAF6 E3 ligase activity with a small-molecule inhibitor combats autoimmunity. J Biol Chem.

[33] Ruzek MC, Huang L, Zhang TT (2018). Dual blockade of interleukin-1beta and interleukin-17A reduces murine arthritis pathogenesis but also leads to spontaneous skin infections in nonhuman primates. J Pharmacol Exp Ther.

[34] Generali E, Bose T, Selmi C, Voncken JW, Damoiseaux J (2018). Nature versus nurture in the spectrum of rheumatic diseases: classification of spondyloarthritis as autoimmune or autoinflammatory. Autoimmun Rev.

[35] Babaie F, Hasankhani M, Mohammadi H (2018). The role of gut microbiota and IL-23/IL-17 pathway in ankylosing spondylitis immunopathogenesis: new insights and updates. Immunol Lett.

[36] Chen DY, Lin CC, Chen YM, Chao YH, Yang DH (2017). Dextromethorphan exhibits anti-inflammatory and immunomodulatory effects in a murine model of collagen-induced arthritis and in human rheumatoid arthritis. Sci Rep.

[37] Luo Z, Yu G, Lee HW (2012). The Nedd8-activating enzyme inhibitor MLN4924 induces autophagy and apoptosis to suppress liver cancer cell growth. Cancer Res.

[38] te Boekhorst BC, Jensen LB, Colombo S (2012). MRI-assessed therapeutic effects of locally administered PLGA nanoparticles loaded with anti-inflammatory siRNA in a murine arthritis model. J Control Release.

[39] Li R, Wang B, He CQ (2015). Upregulation of fibroblast growth factor 1 in the synovial membranes of patients with late stage osteoarthritis. Genet Mol Res: GMR.

[40] Caselli G, Bonazzi A, Lanza M (2018). Pharmacological characterisation of CR6086, a potent prostaglandin E2 receptor 4 antagonist, as a new potential disease-modifying anti-rheumatic drug. Arthritis Res Ther.

[41] Li C, Chen K, Kang H (2017). Double-stranded RNA released from damaged articular chondrocytes promotes cartilage degeneration via Toll-like receptor 3-interleukin-33 pathway. Cell Death Dis.

